# The clinical features and estimated incidence of MIS-C in Cape Town, South Africa

**DOI:** 10.1186/s12887-022-03308-z

**Published:** 2022-05-02

**Authors:** Claire Butters, Deepthi Raju Abraham, Raphaella Stander, Heidi Facey-Thomas, Debbie Abrahams, Ayodele Faleye, Nazneen Allie, Khushbu Soni, Helena Rabie, Christiaan Scott, Liesl Zühlke, Kate Webb

**Affiliations:** 1 Red Cross War Memorial Children’s Hospital, University of Cape Town, Cape Town, South Africa; 2 Institute of Infectious Disease and Molecular Medicine (IDM), Department of Pathology, Division of Immunology, Faculty of Health Science, University of Cape Town, Cape Town, South Africa; 3 Tygerberg Hospital, Stellenbosch University, Cape Town, South Africa; 4 Lagos State University Teaching Hospital, Ikeja, Lagos, Nigeria; 5 Division of Cardiology, Department of Medicine, Groote Schuur Hospital, Cape Town, South Africa; 6 Faculty of Health Sciences, Cape Heart Institute (CHI), University of Cape Town, Cape Town, South Africa; 7 Crick African Network, Francis Crick Institute, London, UK

**Keywords:** MIS-C, SARS-CoV-2, Paediatrics, Low-middle income countries, Global health, Epidemiology, Incidence

## Abstract

**Background:**

Multisystem inflammatory syndrome is a severe manifestation of SARS-CoV-2 in children. The incidence of MIS-C after infection is poorly understood. There are very few cohorts describing MIS-C in Africa despite MIS-C being more common in Black children worldwide.

**Methods:**

A cohort of children with MIS-C and healthy children was recruited from May 2020 until May 2021 from the two main paediatric hospitals in Cape Town, South Africa. Clinical and demographic data were collected, and serum was tested for SARS-CoV-2 antibodies. The incidence of MIS-C was calculated using an estimation of population exposure from seroprevalence in the healthy group. Summary data, non-parametric comparisons and logistic regression analyses were performed.

**Results:**

Sixty eight children with MIS-C were recruited with a median age of 7 years (3.6, 9.9). Ninety seven healthy children were recruited with a 30% seroprevalence. The estimated incidence of MIS-C was 22/100 000 exposures in the city in this time. Black children were over-represented in the MIS-C group (62% vs 37%, *p* = 0.002). The most common clinical features in MIS-C were fever (100%), tachycardia (98.5%), rash (85.3%), conjunctivitis (77.9%), abdominal pain (60.3%) and hypotension (60.3%). The median haemoglobin, sodium, neutrophil count, white cell count, CRP, ferritin, cardiac (pro-BNP, trop-T) and coagulation markers (D-dimer and fibrinogen) were markedly deranged in MIS-C. Cardiac, pulmonary, central nervous and renal organ systems were involved in 71%, 29.4%, 27.9% and 27.9% respectively. Ninety four percent received intravenous immune globulin, 64.7% received methylprednisolone and 61.7% received both. Forty percent required ICU admission, 38.2% required inotropic support, 38.2% required oxygen therapy, 11.8% required invasive ventilation and 6% required peritoneal dialysis. Older age was an independent predictor for the requirement for ionotropic support (OR = 1.523, CI 1.074, 2.16, *p* = 0.018). The median hospital stay duration was 7 days with no deaths.

**Conclusion:**

The lack of reports from Southern Africa does not reflect a lack of cases of MIS-C. MIS-C poses a significant burden to children in the region as long as the pandemic continues. MIS-C disproportionately affects black children. The clinical manifestations and outcomes of MIS-C in this region highlight the need for improved surveillance, reporting and data to inform diagnosis and treatment.

**Supplementary Information:**

The online version contains supplementary material available at 10.1186/s12887-022-03308-z.

## Introduction

Multisystem inflammatory syndrome in children (MIS-C) emerged in 2020 as a serious paediatric manifestation of the severe acute respiratory syndrome coronavirus 2 (SARS-CoV-2) and has been described throughout the world [1–4]. MIS-C is reported in less resourced settings [5–8] but there are very few reports from Africa [9–11]. The incidence of a child developing MIS-C after exposure to SARS-CoV-2 is not well known as exposure rates to SARS-CoV-2 in children are largely unknown. Here, we describe a comprehensive cohort of children with MIS-C in a single city in South Africa (SA) along with a group of healthy children from the same population, exposed to SARS-CoV-2 during a similar time. We describe the clinical features of MIS-C in this population and provide a local serology-based estimation of incidence of MIS-C after exposure to SARS-CoV-2.

With widespread vaccination in the more-resourced world, it is expected that there will be fewer cases of MIS-C as the pandemic abates. Low and middle-income countries (LMICs) in Africa have the challenge of delayed vaccination, poor vaccine coverage and a relatively larger proportion of younger people in the population [12]. In every cohort reported to date, black children are disproportionately affected by MIS-C [1, 2, 13, 14] with an estimation of a nearly six times higher risk of developing MIS-C as compared to white children [15]. We expect that MIS-C will persist in Africa after cases decrease elsewhere. Therefore, it is important to estimate the incidence of MIS-C after SARS-CoV-2 infection and describe the clinical features of MIS-C in this population.

## Methods

### Participants

#### MIS-C

Children under the age of 18 years old admitted to the two main paediatric referral hospitals in Cape Town; the Red Cross War Memorial Children’s Hospital (RXH) or Tygerberg Academic Hospital (TBH) between 7 May 2020 and 31 March 2021 with confirmed MIS-C as per the WHO criteria [16] were offered recruitment with consent. Twenty three cases were previously reported in a letter in August 2020 [9], and four patients have been reported in a renal case series [17].

#### Healthy participants

From 17 August 2020 to 31 May 2021 healthy children attending RXH for elective surgery were enrolled with consent and blood was taken and tested for the presence of SARS-CoV-2 antibodies [18]. Healthy children were only included if they had no underlying chronic disease and were undergoing elective procedures for disorders not related to underlying disease, for example: circumcision, squint repair and fracture revision.

No children in the Western Cape had been vaccinated at the time of recruitment.

Race, sex, and comorbidity data were collected for all patients as recorded in the clinical notes. SARS-CoV-2 contact was defined as confirmed (contact with a person with a positive SARS-CoV-2 PCR test in the preceding 6 weeks), suspected (contact with an unwell person with any fever or flu-like symptoms in the preceding 6 weeks) or none. The presence or absence of clinical features over the entire admission as recorded by clinicians was entered directly to an online case report data sheet. These features included fever, rash, diarrhoea, headache, abdominal pain, conjunctivitis, and arthritis.

The presence or absence of the following clinical signs was collected (with definitions):

- hypotension (defined as blood pressure less than the established range for age recorded > 3 times in 24 h or persistent for more than 1 h)
- tachycardia (defined as heart rate greater than the established range for age)

Organ involvement was defined as any documented involvement of either the cardiac, renal, pulmonary, or central nervous systems (CNS). In children who had received an echocardiogram, the minimum ejection fraction (EF) and presence of a pericardial effusion, any valvular disease or coronary artery aneurysms was collected as recorded in the clinical notes by the cardiologist performing the echocardiogram.

The following blood test results were collected from each admission:

- Maximum: C-reactive protein (CRP), white cell count (WCC), neutrophil count, creatinine, urea, alanine transaminase (ALT), aspartamine transaminase (AST), troponin T (Trop-T), Pro-beta natriuretic protein (BNP), ferritin, D-Dimer, fibrinogen, lactate dehydrogenase (LDH).
- Minimum: haemoglobin (Hb), sodium, albumin, platelets, lymphocyte count.

Treatment with antibiotics, intravenous immune globulin (IVIG) and glucocorticoids was recorded. Intensive care unit (ICU) admission, supplementary oxygen therapy, invasive ventilation, inotropic blood pressure support or dialysis treatments and total duration of admission was recorded.

In a subset of children from RXH only (41/68), daily data was captured from the notes to estimate the median day of admission after symptom onset, median day of treatment and compare the duration of fever related to treatment. Results from microbiological laboratory tests (blood and stool cultures and other viral tests) were recorded in the 41 patients from RXH only.

Children were included after informed consent was obtained and this study was approved by the Human Research Ethics Committee of RXH, TBH and the University of Cape Town (Ethics approval: UCT HREC 112/2012, 599/2020; RXH RCC240/WC_202008_113; TBH N20/07/041; N20/04/013).

### Statistical analysis

Medians and interquartile ranges were reported due to the nonparametric distribution of these data. Comparison of proportions were measured using Chi squared or Fisher exact test where appropriate and continuous data were summarised using an independent sample t-test or Mann–Whitney U test where appropriate. Analysis of data was performed using SPSS V.27.

## Results

### Demographics

**Fig. 1:**
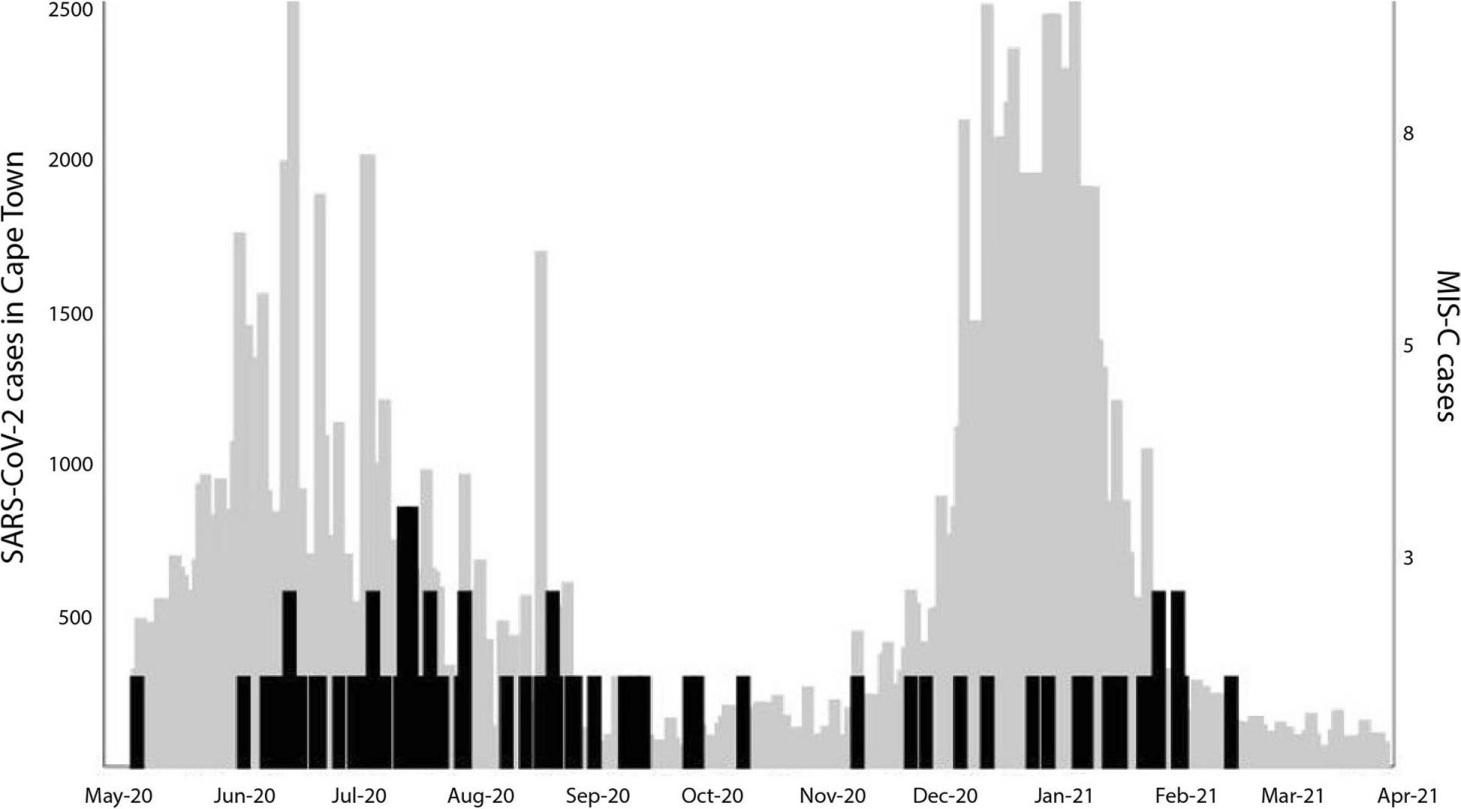
MIS-C in Cape Town, South Africa. 68 children with MIS-C were recruited at the Red Cross and Tygerberg Children’s Hospitals between 7 May 2020 and 30 March 2021. Black bars indicate MIS-C cases at these two main paediatric referral centers while grey bars indicate the daily number of SARS-CoV-2 cases in the City of Cape Town, South Africa [19]. As per the National Institute of Communicable Diseases’s definition, the first two waves of SARS-CoV-2 in the city occurred from 3 May 2020 – 16 August 2020, and 8 November 2020 – 7 February 2021, with the third wave starting on 23 May 2021

Sixty-eight children with MIS-C [16] were recruited (41 from RXH and 27 from TBH), reflecting cases collected up to 6 weeks after the end of the second wave of SARS-CoV-2 in the city (Fig. 1). The median age of children with MIS-C was 7 years (Interquartile range (IQR) 3.6, 9.9) with no sex bias (47.1% female) and 42/68 (62%) were Black African (Table 1) The youngest child with MIS-C was 2 months old and the oldest was 14 years old, reflecting the arbitrary local admission age cut-off for paediatric services. Pre-existing conditions were recorded in 13/68 (19.1%) of children with MIS-C (Supp. Table 1).

**Table 1:** Demographics

	MIS-C	Healthy children	
Median age (years) (IQR)	7.0 (3.6, 9.9)	5.4 (2.4, 9.0)	*p* = 0.379
Female (%)	32/68 (47.1)	31/97 (31.9)	*p* = 0.092
Black African (%)	42/68 (61.8)	36/97 (37.1)	*p* = 0.002
South African Coloured (%)	26/68 (38.2)	61/96 (62.8)	

In order to estimate seroprevalence among healthy children during the first 2 waves in the city, 97 healthy children were recruited. The time periods are slightly different for the recruitment of healthy children and those with MIS-C as elective surgeries were cancelled during both waves, so that recruitment of healthy children lagged slightly. Ultimately, all healthy children were recruited prior to the 3^rd^ wave, so that seroprevalence most likely reflects infection during the first 2 waves (Supp. Figure 1). Healthy children had a median age of 5.5 years (IQR 2.4, 9) and 31% were female. There was no difference in age or sex in healthy children as compared to children with MIS-C (Table 1). A lower proportion of healthy children were Black African as compared to children with MIS-C (37.1% vs 62%, *p* = 0.002).

All healthy children were nasal swab SARS-CoV-2 polymerase chain reaction (PCR) negative and 29/68 (30%) had SARS-CoV-2 antibodies in serum (exposed) (Supp. Figure 1). Healthy exposed children tended to be younger than healthy unexposed children (3.3 years vs 6.1 years, *p* = 0.066) but there was no difference in sex (41% vs 30% female, *p* = 0.318) or ethnicity (45% vs 34% Black, *p* = 0.304) by exposure.

### Incidence

The estimated population of children under 14 in Cape Town is 1 044 963 [20], giving an estimated population incidence of 6.6 MIS-C cases per 100 000 children aged less than 14 in the city during the first 2 SARS-CoV-2 waves. Applying the 30% seroprevalence seen in the group of healthy children, we estimate that 313 488 children may have been exposed to SARS-CoV-2 in the city during the first 2 waves. This allows us to estimate an incidence of 22 cases of MIS-C per 100 000 SARS-CoV-2 infections in children younger than 14 in the city up until this date.

### Clinical features of MIS-C

Twenty three percent of children with MIS-C (14/61) had a confirmed SARS-CoV-2 contact, 18% (11/61) had a suspected contact and 59% (36/61) had no contact reported (Supp. Table 2). Ninety one percent had a positive SARS-CoV-2 antibody test and 14.7% had a positive PCR test at the time of admission (Supp. Table 2). None of the children had received prior SARS-CoV-2 vaccination.

The most common clinical features in children with MIS-C were fever (100%), tachycardia (98.5%), rash (85.3%), conjunctivitis (77.9%), abdominal pain (60.3%) and hypotension (60.3%) (Table 2). The average total duration of fever was 6 days (IQR 4, 8). The IQR of the median minimum Hb and sodium were less than the local normal range in MIS-C (Table 2). The IQR of the median maximum neutrophil count, WCC, CRP, ferritin, cardiac (pro-BNP, trop-T) and coagulation markers (D-dimer and fibrinogen) were higher than the local normal range (Table 2). The laboratory tests that were most frequently abnormal as compared to the local normal range were CRP (100%), sodium (98%), ferritin (98%), D-Dimer (98%), haemoglobin (88%) and neutrophil count (88%). Fifty three percent (36/68) of children with MIS-C met the clinical criteria for Kawasaki Disease [21]. Thirteen percent (9/68) met the criteria for macrophage activation syndrome (MAS) [22] but no patients had bone marrow assays to demonstrate haemophagocytosis and none had persistent secondary MAS subsequent to resolution of MIS-C.

**Table 2:** Clinical findings

Signs and Symptoms	Count (%) *n* = 68^a^	Laboratory findings (normal range)		Median *n* = 68^a^	N abnormal (%)
Fever	68 (100)	Minimum	Sodium (136–145 mmol/L)	^b^129 (127, 131)	67 (98)
Tachycardia	67 (98.5)		Platelets (180–440 × 10^9^/L)	189 (134, 275)	32 (47)
Rash	58 (85.3)		Hb (11.8–14.6 g/dL)	^b^9.7 (8.4, 10.8)	60 (88)
Conjunctivitis	53 (77.9)		Albumin (29–42 g/L)	27 (25, 30) *n* = 63	44 (70)
Abdominal pain	41 (60.3)		Lymphocyte count (1.90–4.30 × 10^9^/L)	1.16 (0.75, 2.28) *n* = 67	44 (66)
Hypotension	41 (60.3)	Maximum	CRP (< 10 mg/L)	^b^242 (140, 308) *n* = 67	67(100)
Diarrhoea	40 (58.8)		Troponin-T (< = 14 ng/L)	^b^38 (16, 72) *n* = 45	34 (75)
Mucositis	19/41 (46.3)		pro-BNP (< 450 ng/L)	^b^5785 (737, 19,898) *n* = 62	50 (81)
Arthritis	19 (27.9)		Neutrophil count (1.7–5.0 × 10^9^/L)	^b^11.99 (7.30, 18.49) *n* = 67	59 (88)
Headache	18 (26.4)		WCC (3.90–10.20 × 10^9^/L)	^b^16.60 (12.54, 24.74)	57 (84)
**Organ Involvement**			Ferritin (7–84 ng/L)	^b^643 (317, 1022) *n* = 65	64 (98)
Lung	20 (29.4)		D-dimer (0.00–0.25 mg/L)	^b^2.60 (1.29, 4.91) *n* = 59	58 (98)
CNS	19 (27.9)		Fibrinogen (2.0–4.0 g/L)	^b^5.3 (4.5, 6.4) *n* = 57	48 (84)
Renal	19 (27.9)		Urea (1.4–5.7 mmol/L)	6.8 (4.2, 12.7)	40 (59)
**Cardiac**			Creatinine (30–48 umol/L)	47 (35, 76)	33 (49)
Mitral regurgitation	25 (36.8)		AST (0–41 U/L)	46 (31, 61) *n* = 61	34 (73)
Pericardial effusion	12 (17.6)		ALT (5–25 U/L)	33 (22, 55) *n* = 64	44 (69)
Coronary artery aneurysm	4 (5.9)		Maximum LDH (110–295 U/L)	325 (279, 419) *n* = 25	15 (60)

*CNS* Central nervous system, *CRP* C-reactive protein, *Hb* haemoglobin, *WCC* white cell count, *ALT* alanine transaminase, *AST* aspartamine transaminase, *pro-BNP* Pro-beta natriuretic protein, *LDH* lactate dehydrogenase

^a^*n* = 68 unless specified

^b^Interquartile range of median estimate is outside of normal range of laboratory test

Pulmonary, CNS and renal organ systems were involved in 29.4%, 27.9% and 27.9% respectively (Table 2). Seventy one percent had cardiac involvement which included pericardial effusions (17.6%), mitral regurgitation (36.8%) and coronary artery aneurysms (5.9%). The estimated median EF was 47% (IQR 39, 60). Children with MIS-C and hypotension had a lower EF compared to non-hypotensive patients (*p* < 0.0001). Eighty five percent of children with MIS-C had gastrointestinal symptoms, including abdominal pain (60.3%), diarrhoea (58.8%) and vomiting (10/41). One child underwent a negative laparotomy for a suspected appendicitis. Nine of 41 children with microbiology lab data (22%) had evidence of other infections during admission. Of these, the majority were judged to be hospital acquired infections or contaminants, but it was determined that two children had a true co-infection in addition to MIS-C, both with evidence of *Escherichia coli* (*E. coli*) urinary tract infections (UTI) (Supp. Table 3).

**Fig. 2:**
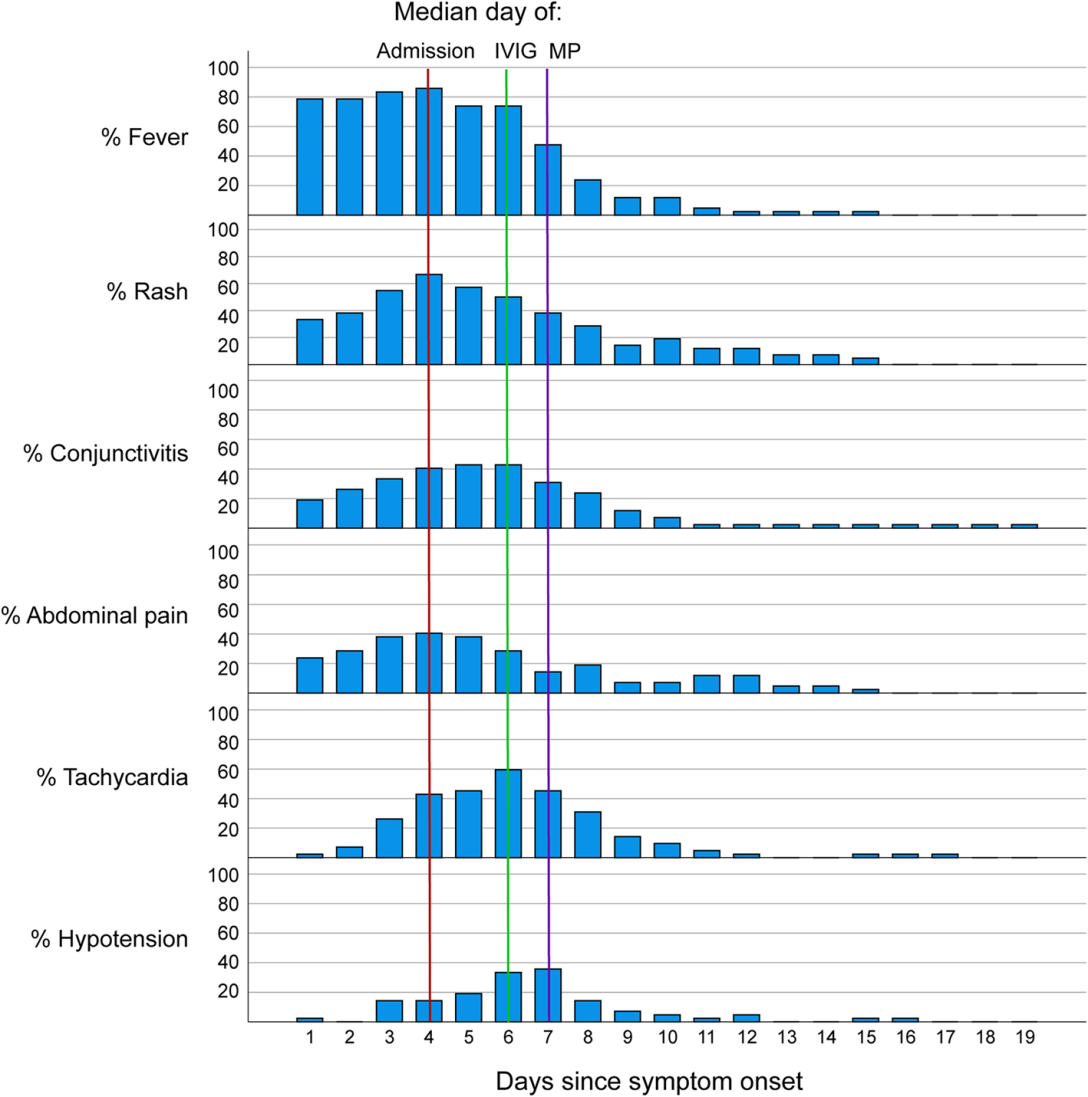
Resolution of clinical features. Of 41 children at the Red Cross Children's Hospital, daily clinical data was captured. The percentage of children with the clinical features of the cohort is reflecting for each day since the onset of symptoms to demonstrate the average kinetics of resolution with time in relation to admission and treatment. The red line indicates the median day of admission (day 4), the green line indicates the median day of receiving IVIG (day 6) and purple line indicates the median day of receiving methylprednisolone (day 7). IVIG- Intravenous immune globulin. MP- Methylprednisolone

A single antibiotic was administered to 94.1% of children, and 23.5% received an additional antibiotic during their admission. The median day of initiation of antibiotics was day 1 (IQR 1, 1) of admission (Supp. Table 4). The most frequently administered MIS-C treatment was IVIG; 94.1% of children received at least one dose and 8.8% required a second dose. Intravenous methylprednisolone was given to 64.7% of children. Twenty-two (32.4%) children received IVIG only, two (2.9%) received methylprednisolone only, 42 (61.7%) received both IVIG and methylprednisolone and two (2.9%) received neither IVIG nor methylprednisolone. Six percent (4/68) of children received an IL-6 inhibitor (tocilizumab). The median day of admission was day 4 (IQR 4, 6) after symptom onset. The median day of treatment with IVIG was day 2 after admission (IQR 2, 4) and the median day of treatment with methylprednisolone was day 3 after admission (IQR 3, 5). Fever, tachycardia and hypotension remitted soon after treatment in the majority of patients, although the presence of a rash persisted for longer in some patients (Fig. 2).

Children who received both IVIG and methylprednisolone were more frequently admitted to ICU compared to those that received IVIG alone (24/42 vs 3/22, *p* = 0.001). Children who received IVIG and methylprednisolone vs IVIG alone had no difference in the median maximum CRP (183 mg/L vs 246 mg/L, *p* = 0.28), duration of fever (6 days in each group) or duration of hospital stay (7 vs 6 days, *p* = 0.142).

Of all 68 children, 27 (39.7%) required ICU admission, 26 (38.2%) required inotropic blood pressure support, 26 (38.2%) required oxygen therapy, 8 (11.8%) required invasive ventilation and 4 (6%) required peritoneal dialysis. The median hospital stay duration was 7 days (IQR 6, 10). No patients died at either hospital.

Inotropic support was further explored as a proxy for severity. The median age of children with MIS-C who required inotropic support was higher than those who did not (8 years (IQR 7, 11) vs 4 years (IQR 1.4, 9), *p* = 0.001). Equally, CRP (*p* = 0.02), troponin T (*p* = 0.05), Hb (*p* = 0.05), platelets (*p* = 0.41) and pro-BNP (*p* < 0.001) all independently associated with the need for inotropic support. A logistic regression model showed that when controlling for CRP, pro-BNP, Trop T, Hb and platelets, age was a notable predictor of the need for inotropic support (OR = 1.523, CI 1.074, 2.16, *p* = 0.018).

## Discussion

The lack of data from Southern Africa may lead to the false conclusion that MIS-C is rare in the region and we hope that these data highlight the burden that African children face from MIS-C, while providing valuable clinical and epidemiological observations in the global discovery of this disease.

Cape Town is a relatively well-resourced city in the region, with an established research infrastructure which allows for the rapid and comprehensive documentation of cases of MIS-C in the two main paediatric state hospitals in the city. Although MIS-C was declared a notifiable medical condition in South Africa in September 2020 [23], the uptake of national notifications has been poor and the true burden of MIS-C in the country and region is unknown. Due to a tight network of paediatricians in the city, with 2 main referral hospitals, this cohort represents the majority of children with MIS-C in the city but may exclude a small number of milder cases treated in primary care centres, in the private sector or children with severe MIS-C who presented with shock and died before diagnosis.

The incidence of MIS-C after infection with SARS-CoV-2 has been difficult to define globally as it has been difficult to accurately estimate the number of children exposed to SARS-CoV-2 in different regions. Reported numbers of SARS-CoV-2 infections in most paediatric populations are inaccurate as children are commonly asymptomatic and may not be tested. In the US, access to testing varied widely between regions over the course of the pandemic, resulting in poor correlation between regional numbers of paediatric SARS-CoV-2 cases and MIS-C cases [4]. Payne et al. attempted to overcome this problem by using regional multipliers to account for the various causes of under-reporting of SARS-CoV-2 to estimate the total number of children exposed to the virus per state in the US [15, 24]. Adjusting for race, age and jurisdiction, they estimate a total incidence of 31.5 cases of MIS-C per 100 000 SARS-CoV-2 infections in people younger than 21 years old in the US, with regional unadjusted estimates varying between 13.1/100 000 in New York and 77.9/100 000 in Michigan.

Here, we use the 30% seroprevalence observed in a group of healthy children from the same area and time of exposure as the children with MIS-C, to estimate an incidence of 22 cases per 100 000 SARS-CoV-2 infections in children less than 14 years old in the city. We do not adjust for age or ethnicity, but black children and children under 15 accounted for the highest incidence in the US [15], making our relatively lower incidence in a younger population, with a high proportion of black children surprising. As mentioned, we may slightly under-estimate the true number of cases of MIS-C in the city—if we assume that at worst, we underestimate cases by 20%, we still get a relatively conservative estimated incidence of 26/100 000.

We argue that serology gives a more accurate estimation of the denominator - the number of children who have been infected with SARS-CoV-2 - than other studies which most likely explains the lower incidence reported here. This is a small sample of healthy children making the generalisability of the seroprevalence in this sample a weakness. Equally, these children were attending the hospital, which may introduce bias, although this was limited by only recruiting children undergoing elective procedures not associated with underlying inflammatory, immune or other chronic illnesses.

As future waves produce multiple infections, it will not be possible to assume that seropositivity associates with a single infection. In the absence of widespread longitudinal community studies which link cases of MIS-C to single (often asymptomatic) infections, we argue that this cohort, in a defined geographic area and time may yield our best (although imperfect) estimation of incidence after infection.

This cohort follows the established global trend of MIS-C being more common in Black children. We show no evidence of difference in SARS-CoV-2 exposure in Black children in the healthy cohort, arguing that differences in exposure cannot account for these differences. Although we do not account for many other racial and historical factors relevant to our context, such as access to care, burden of disease and nutrition, we emphasise the possibility that Black children may be at an inherently higher risk of MIS-C and deserve extra vigilance. The demographic distribution of Cape Town is not reflective of the rest of the country where 80% of the population is Black African leading us to believe that there is likely a higher burden of MIS-C throughout the country than is described here, and that efforts to improve monitoring and reporting are urgently warranted.

The clinical disease presentation in this cohort is similar to other reported cohorts with rash, abdominal pain, conjunctivitis, tachycardia and hypotension being common. The proportion of children with confirmed exposure to MIS-C is similar to the US (22% vs 24.7%) [2]. Serum sodium, CRP, ferritin, haemoglobin, neutrophil count, cardiac markers and coagulation markers were markedly deranged in children with MIS-C. Cardiac involvement was common, but not universal in this cohort with a relatively lower rate of coronary artery aneurysms than other cohorts [2]. The relationship between a low EF and hypotension confirms the myocardial nature of the disease.

Treatment efficacy cannot be inferred from these data, which reflect local treatment recommendations where children who required ICU admission received both glucocorticoids and IVIG. There are no prospective treatment data for MIS-C and retrospective propensity matched analyses are conflicting as to whether IVIG works better alone or in conjunction with glucocorticoids [25–27]. IVIG is an expensive and inaccessible medication in many regions in Africa. The Best Available Treatment Study [26] is the only study to date to include patients who received glucocorticoids alone, and did not show superiority of IVIG alone as compared to glucocorticoids alone, although the dose and route of administration of glucocorticoids was not specified. Therefore, prospective randomized treatment studies designed for lower-middle-income settings are urgently needed.

Approximately one third of cases had any involvement of the brain, kidneys or lungs and nearly half required ICU admission, highlighting the severity of this disease and the requirement for a skilled and multi-disciplinary team. Older age (> 7 years) emerged as an independent predictor of severity (as defined by the requirement for inotropic support), with important clinical utility.

In conclusion, we provide a detailed description of a cohort of children with MIS-C in a Southern African setting, with the first seroprevalence based estimation of the incidence of MIS-C after SARS-CoV-2 exposure. These data highlight that MIS-C is a severe disease, causing significant illness in children from this region and most likely others in Africa. We emphasise that the burden of MIS-C in children from this region is under-represented in global data. With the inequality of SARS-CoV-2 vaccination, we expect that MIS-C will persist as a threat to African children. There is an urgent need to improve surveillance and perform research in the region to better inform the diagnosis and management of these children.

## Supplementary Information

**Additional file 1: Figure S1.** Seroprevalence of SARS-CoV-2 in healthy children. 97 children presenting to RXH for elective surgeries that were otherwise well were recruited between August 2020 and May 2021. Twenty-nine of these healthy children had antibodies to SARS-CoV-2 (healthy exposed) with an estimated seroprevalence of 30% during this period.

**Additional file 2: Table S1.** Pre-existing conditions in children with MIS-C.

**Additional file 3: Table S2.** SARS-CoV-2 exposure.

**Additional file 4: Table S3.** Evidence of infection.

**Additional file 5: Table S4.** Treatment.

## Data Availability

Data represents detailed clinical data from minors. Therefore, due to privacy and ethical concerns, data are not being made publicly available, however may be available upon request from the corresponding author.
