# Supplementary material for: The clinical features and estimated incidence of MIS-C in Cape Town, South Africa
Source: BMC Pediatr. 2022 May 2;22:241. doi: 10.1186/s12887-022-03308-z (PMC9059902; doi:10.1186/s12887-022-03308-z)
Supplement: Supplementary file 1 — Additional file 1: Figure S1. Seroprevalence of SARS-CoV-2 in healthy children. 97 children presenting to RXH for elective surgeries that were otherwise well were recruited between August 2020 and May 2021. Twenty-nine of these healthy children had antibodies to SARS-CoV-2 (healthy exposed) with an estimated seroprevalence of 30% during this period. [file 12887_2022_3308_MOESM1_ESM.docx]

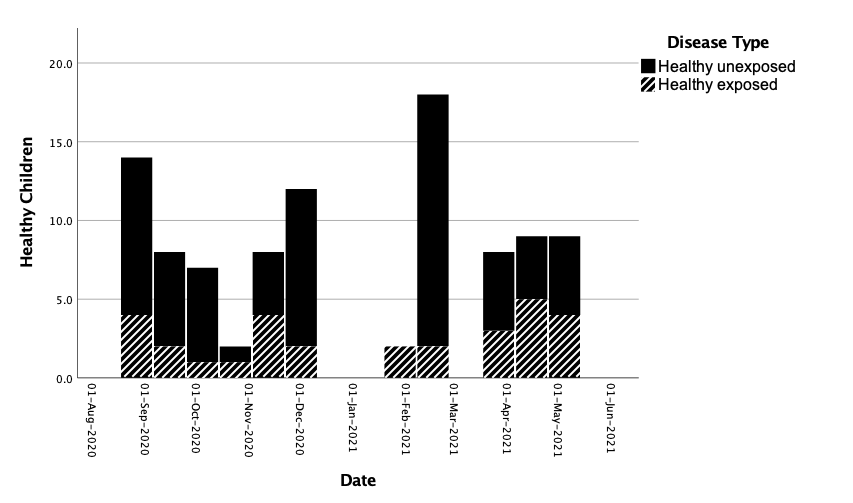


**Supplementary Figure 1: Seroprevalence of SARS-CoV-2 in healthy children.** 97 children presenting to RXH for elective surgeries that were otherwise well were recruited between August 2020 and May 2021. Twenty-nine of these healthy children had antibodies to SARS-CoV-2 (healthy exposed) with an estimated seroprevalence of 30% during this period.
