# Supplementary material for: The clinical features and estimated incidence of MIS-C in Cape Town, South Africa
Source: BMC Pediatr. 2022 May 2;22:241. doi: 10.1186/s12887-022-03308-z (PMC9059902; doi:10.1186/s12887-022-03308-z)
Supplement: Supplementary file 2 — Additional file 2: Table S1. Pre-existing conditions in children with MIS-C. [file 12887_2022_3308_MOESM2_ESM.docx]

Supplementary Table 1: Pre-existing conditions in children with MIS-C

|  | Count |
| --- | --- |
| Acute myeloid leukemia | 2 |
| Eczema | 1 |
| Epilepsy | 1 |
| Hereditary exostosis | 1 |
| HIV exposed in utero and subsequently HIV negative | 2 |
| Obesity | 5 |
| Perthes disease | 1 |
