# Supplementary material for: The clinical features and estimated incidence of MIS-C in Cape Town, South Africa
Source: BMC Pediatr. 2022 May 2;22:241. doi: 10.1186/s12887-022-03308-z (PMC9059902; doi:10.1186/s12887-022-03308-z)
Supplement: Supplementary file 3 — Additional file 3: Table S2. SARS-CoV-2 exposure. [file 12887_2022_3308_MOESM3_ESM.docx]

Supplementary Table 2: SARS-CoV-2 exposure

|  | | Count (%) |
| --- | --- | --- |
| PCR positive | | 10/ 68 (14.7) |
| Antibody positive | Any serology positive | 62 (91.2) |
|  | Total IG to Nucleocapsid* | 28/34 (82.4) |
|  | IgG to Nucleocapsid | 20/22 (90.9) |
|  | IgG to Spike | 37/39 (94.9) |
|  | IgA to Spike | 24/25 (96.0) |
| SARS-CoV-2 contact | Confirmed | 14 (23.0) |
|  | Suspected | 11 (18.0) |
|  | None | 36 (59.0) |

* 22 from TBH only received antibody tests at their follow-up appointment (after 13 October 2020 when serology tests became available).

IgG- Immune globulin G

IgA- Immune globulin A
