# Supplementary material for: The clinical features and estimated incidence of MIS-C in Cape Town, South Africa
Source: BMC Pediatr. 2022 May 2;22:241. doi: 10.1186/s12887-022-03308-z (PMC9059902; doi:10.1186/s12887-022-03308-z)
Supplement: Supplementary file 4 — Additional file 4: Table S3. Evidence of infection. [file 12887_2022_3308_MOESM4_ESM.docx]

Supplementary Table 3: Evidence of infection

| Nosocomial candida | 9yr old with MIS-C requiring ventilation and ICU admission with central line insertion, and peritoneal dialysis. Light growth of candida during ICU stay, most likely hospital acquired. |
| --- | --- |
| Urinary contaminant ESBL E Coli | 3.5yr with MIS-C (rash, fever, conjunctivitis, headache, responded to IVIG and methylprednisolone, raised pro-BNP, D-dimer, and decreased EF). Extended spectrum beta-lactamase producing E.Coli cultured in urine on day of admission. Nil urinary leukocytes or erythrocytes, most likely contaminant. Also had rhinovirus on nasal swab. |
| Nosocomial/contaminant yeast in stool | 14yr old with MIS-C (fever rash conjunctivitis, tachycardia, hypotension and covid exposure requiring with cardiac disease with EF 32%, ICU, inotropes, IVIG and methylprednisolone). Stool culture done on day 9 for ongoing diarrhoea. Light growth of yeast, likely hospital acquired. |
| E.Coli UTI co-infection | 1.5yr old with MIS-C (fever, rash, swollen hands, tachycardia, mucositis, conjunctivitis, raised pro-BNP and D-dimer, received IVIG). On day of admission, had 3+ leukocytes and E coli UTI. On review decided that likely MIS-C with co-existing UTI. |
| E.Coli UTI co-infection, nosocomial candida and enterococcus | 8yr old with MIS-C (abdominal pain, shock, rash, conjunctivitis, requiring ICU, peritoneal dialysis, inotropic support). Cultured E coli grown in urine from day 1. Most likely coexisting UTI. Subsequently cultured enterococcus and candida UTI during ICU admission. Most likely hospital acquired/contaminant. |
| Hospital acquired enterococcus | 7yr old female with MIS-C (fever, shock, rash, abdominal pain, requiring inotropes, ICU admission, markedly raised pro-BNP). Had an enterovirus PCR positive on day 9 of ICU admission with negative result 4 days earlier. |
| Multiple ICU acquired nosocomial infections | 6-month-old with MIS-C (shock, tachycardia, fever, multisystem disease, markedly raised pro-BNP, D-dimers and positive covid serology) with prolonged admission and multiple nosocomial infections in ICU during prolonged stay including acinetobacter baumanii, klebsiella pneumonia candida and rhinovirus. All judged to be hospital acquired. |
| Contaminant staphylococcus aureaus | 10 year old with MIS-C (fever, headache, conjunctivitis, rash, arthritis, raised D-Dimers, mucositis, responded to IVIG and methylprednisone) had positive blood culture from day of admission (19 hours) with staphylococcus aureas, penicillin resistant, cloxacillin sensitive. Judged to be a contaminant by treating clinicians as did not fit clinical picture and antibiotics (ceftriaxone) stopped on day 2. Subsequent cultures negative. |
| Nosocomial rhinovirus | 7-year-old with severe MIS-C requiring ICU, dialysis and ionotropic support had rhinovirus positive nasal swab test during ICU admission – judged to be nosocomial and incidental. |
