# Supplementary material for: The clinical features and estimated incidence of MIS-C in Cape Town, South Africa
Source: BMC Pediatr. 2022 May 2;22:241. doi: 10.1186/s12887-022-03308-z (PMC9059902; doi:10.1186/s12887-022-03308-z)
Supplement: Supplementary file 5 — Additional file 5: Table S4. Treatment. [file 12887_2022_3308_MOESM5_ESM.docx]

Supplementary Table 4: Treatment

|  | | MIS-C  Count (%) |
| --- | --- | --- |
| 1st line antibiotics | | 64 (94.1) |
| 2nd line antibiotics | | 16 (23.5) |
| 1st dose IVIG  n=64 | 1mg/kg | 10 (15.6) |
|  | 2mg/kg | 54 (84.4) |
| 2nd dose IVIG  n=6 | 1mg/kg | 2 (33.3) |
|  | 2mg/kg | 4 (66.7) |
| Methylprednisolone total dose  n=44 | 2mg/kg | 1 (2.3) |
|  | Not reported | 2 (4.5) |
|  | 15mg/kg | 2 (4.5) |
|  | 30mg/kg | 38 (86.4) |
|  | 110mg/kg | 1 (2.3) |
| Oral prednisone | | 44 (64.7) |
| Dexamethasone | | 6 (8.8) |
| Tocilizumab | | 4 (5.9) |
